# Multi-Locus Sequence Analysis Reveals Diversity of the Rice Kernel Smut Populations in the United States

**DOI:** 10.3389/fmicb.2022.874120

**Published:** 2022-05-04

**Authors:** Sabin Khanal, Sanjay Antony-Babu, Shankar P. Gaire, Xin-Gen Zhou

**Affiliations:** ^1^Texas A&M AgriLife Research Center, Beaumont, TX, United States; ^2^Department of Plant Pathology and Microbiology, Texas A&M University, College Station, TX, United States

**Keywords:** Rice, kernel smut, Tilletia, *Tilletia horrida*, *Tilletia barclayana*, genetic diversity

## Abstract

Rice (*Oryza sativa*) is the second leading cereal crop in the world and is one of the most important field crops in the US, valued at approximately $2.5 billion. Kernel smut (*Tilletia horrida* Tak.), once considered as a minor disease, is now an emerging economically important disease in the US. In this study, we used multi-locus sequence analysis to investigate the genetic diversity of 63 isolates of *T. horrida* collected from various rice-growing areas across in the US. Three different phylogeny analyses (maximum likelihood, neighbor-joining, and minimum evolution) were conducted based on the gene sequence sets, consisting of all four genes concatenated together, two rRNA regions concatenated together, and only ITS region sequences. The results of multi-gene analyses revealed the presence of four clades in the US populations, with 59% of the isolates clustering together. The populations collected from Mississippi and Louisiana were found to be the most diverse, whereas the populations from Arkansas and California were the least diverse. Similarly, ITS region-based analysis revealed that there were three clades in the *T. horrida* populations, with a majority (76%) of the isolates clustering together along with the 22 *Tilletia* spp. from eight different countries (Australia, China, India, Korea, Pakistan, Taiwan, The US, and Vietnam) that were grouped together. Two of the three clades in the ITS region-based phylogeny consisted of the isolates reported from multiple countries, suggesting potential multiple entries of *T. horrida* into the US. This is the first multi-locus analysis of *T. horrida* populations. The results will help develop effective management strategies, especially breeding for resistant cultivars, for the control of kernel smut in rice.

## Introduction

Rice (*Oryza sativa* L.) is one of the most important crops with a worldwide production of 509 million metric tons annually (FAOSTAT, 2019). Rice provides a major source of energy for more than half of the world population (FAOSTAT, 2019). In 2019, the US rice production was estimated to be 10 million metric tons. Arkansas, California, Louisiana, Mississippi, Missouri, and Texas are the major rice producers in the US (USDA, 2020). Rice kernel smut, caused by *Tilletia horrida* Tak., causes partial or full bunt in rice grains, resulting in a direct reduction in grain yield and quality (Whitney and Cartwright, 2018). Rice kernel smut was first reported in 1896 in Japan (Takahashi, 1896); currently, rice kernel smut is widespread across rice-growing countries and its distribution is expected to be wider than recently reported (Carris et al., 2006).

Average losses from rice kernel smut have been reported around 15%; however, losses as high as 87 and 100% in Pakistan and China have been reported (Biswas, 2003). Major losses from kernel smut are due to the depletion in grain quality with countries restricting the maximum permissible limit for the smutted grains. Milled rice in the US has a restriction of 3% smutted rice (USDA, 2020). Similarly, certified rice seeds in India have a restriction of 0.5% smutted rice grains (Chahal, 2001). Historically kernel smut was considered a minor disease; however, persistent occurrence and frequent outbreaks of the disease in recent years have made kernel smut as one of the most economically important diseases in rice in many countries (Elshafey, 2018; Wang et al., 2019b; Allen et al., 2020; Zhou et al., 2020). In the US, kernel smut occurrence and severity have been on the rise for the past decade and pose a serious threat to the US rice production (Espino, 2019, 2020; Allen et al., 2020; Way and Zhou, 2020). In 2021, severe outbreaks of kernel smut occurred widely across the Texas rice areas and southwest Louisiana, with the percentage of affected panicles ranged up to 50% and the infected kernels ranged up to 20 percent (Zhou et al., 2021a). In states such as Arkansas and California where rice industry is valued as billion-dollar industry, potential economic losses are even higher (USDA, 2020). With continual increase in acreage compounded with the use of susceptible cultivars, kernel smut has also threatened organic rice production in California and Texas, the two leading states in the US organic rice production (Zhou et al., 2021b).

Rice kernel smut is caused by a basidiomycota fungus, belonging to *Tilletia* genus and Tilletiaceae family. More than 80 genera and 4,200 species of smut fungi have been reported as the pathogens to many plant species (Vánky, 1987). Phylogenetically *Tilletia* species have been considered to separate their lines from those of other smut fungi, *Ustilago* and *Sporisorium* (Roux et al., 1998). *Tilletia horrida* forms thick walled dark teliospores which can be present widely on the soil, plant debris, and rice seeds (Carris et al., 2006; Whitney and Cartwright, 2018). Kernel smut taxonomy has been turbulent through the years of many studies. *Tilletia horrida* Tak., was first described by Takahashi in 1896; however, over the years, various authors reclassified the fungus to different genus and species: *T. barclayana* (Bref.) Sacc. & Syd., *Neovossia barclayana* (Bref.), and *Neovossia horrida* (Tak.; Tullis and Johnson, 1952). Through the years, *T. barclayana* and *T. horrida* have been interchangeably used to describe kernel smut of rice. However, a distinction between *T. horrida* and *T. barclayana* has been demonstrated by various molecular and phylogenetic studies (Levy et al., 2001; Carris et al., 2006). Currently, *T. horrida* has been more commonly used to describe kernel smut of rice in the literature (Wang et al., 2015, 2019a,b; Allen et al., 2020).

Molecular phylogeny through multi-locus sequence typing (MLST) offers an excellent means to parse bacterial population structure with the use of housekeeping gene sequences and hence found a rightful reliable place in disease epidemiology (Maiden et al., 1998). MLST characterizes bacterial strain by their unique allelic profiles by measuring the variations in housekeeping genes. MLST provides a discriminatory power to differentiate different bacterial strains. Although use of MLST is less prevalent in mycology, it has also become a useful tool for studying to understand the fungal populations (Taylor and Fisher, 2003). The method represents an important tool to determine the population of fungi that are pathogenic to humans (Bougnoux et al., 2003; Bain et al., 2007) and plants (Kellner et al., 2011; Choi et al., 2013; Sun et al., 2013; Gurjar et al., 2021). MLSA has also been used to study the genetic diversity of various smut fungi (Kellner et al., 2011; Sun et al., 2013; Gurjar et al., 2021; Sedaghatjoo, 2021). Previous phylogenetic studies of *T. horrida* populations have been rare. One phylogenetic study conducted with *T. horrida* isolates collected from seven different provinces in China did not find any genetic variation (Wang et al., 2018). In the current study, we used the MLSA approach to understand the genetic diversity of the *T. horrida* populations in the US. Understanding the genetic diversity will help in designing and improving rice breeding programs to develop new cultivars with improved kernel smut resistance and in developing effective chemical management strategies for control of kernel smut. The results of our multi-gene phylogeny analyses showed, for the first time, the presence of genetic diversity in the rice kernel smut populations in the US, with all the *T. horrida* isolates clustering into four different genetic groups.

## Materials and Methods

### Collection of Isolates

**Figure 1:**
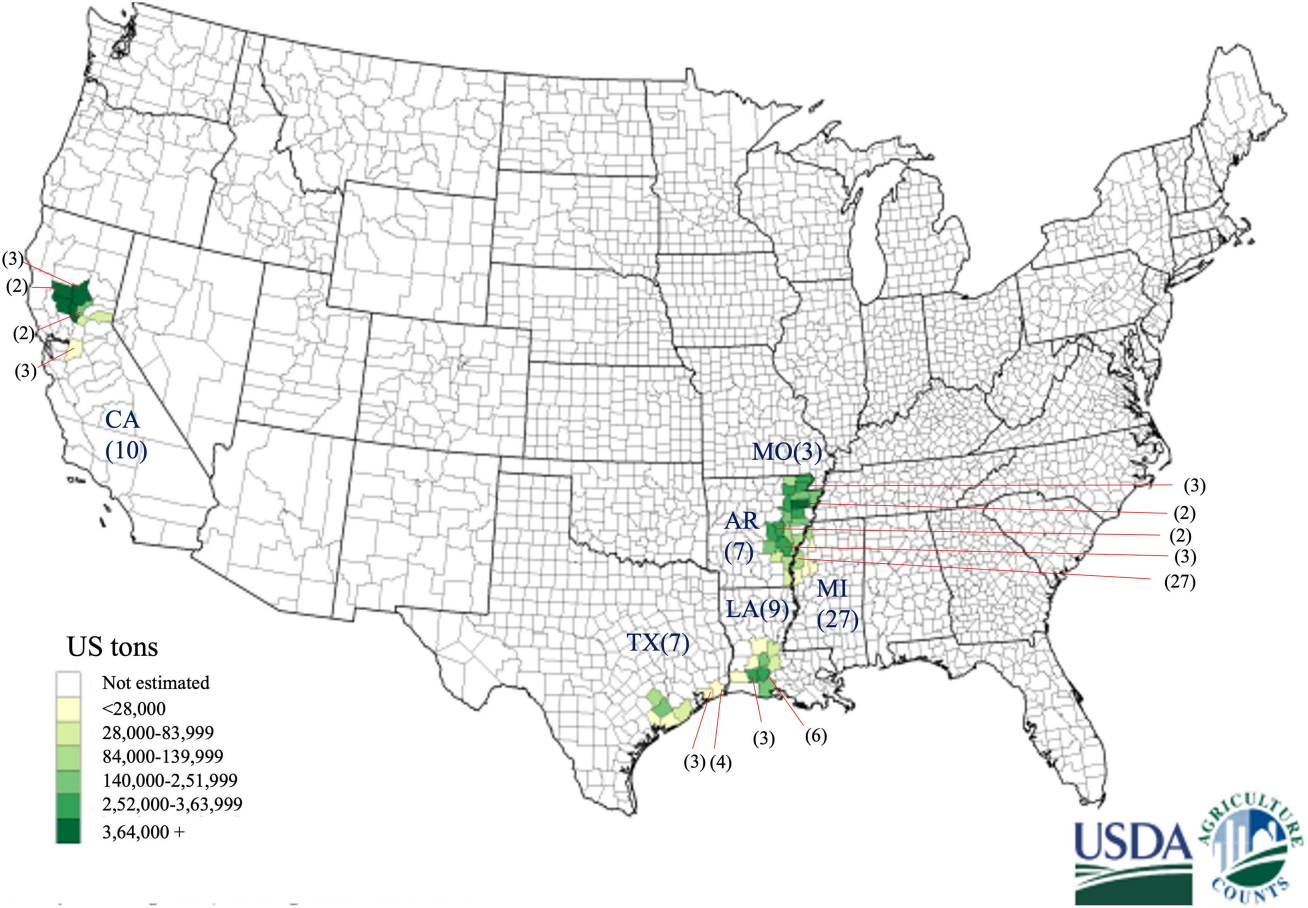
Geographical distribution of 63 *Tilletia horrida* isolates in Arkansas (AR), California (CA), Louisiana (LA), Mississippi (MI), Missouri (MO), and Texas (TX), covering almost all rice-growing areas in the US. Gradient shading areas inside each state represent the rice production in the US in 2019 provided by the USDA National Agricultural statistics services. The number in the parentheses represents the number of isolates from each county pointed by the red arrow.

Rice grain samples were collected in the 2018 and 2019 growing seasons from six major different rice-growing states in the US (Figure 1; Table 1). Rice grain samples showing the symptoms of kernel smut were brought to the Plant Pathology Lab at the Texas A&M AgriLife Research Center, Beaumont, Texas. Sixty-three fungal isolates were isolated from the infected rice grain samples. Putative *T. horrida* were isolated from teliospores in 2% water-agar, based on the procedure described previously (Chahal et al., 1993). Germination of teliospores was visually confirmed under microscope after 3 days of incubation. Primary sporidia that germinated from the single teliospores were transferred to potato dextrose agar (PDA) plates and incubated for growth at 28^o^ C for 14 days. Mycelium was stored in a solution comprising of 2% of sucrose and 20% of glycerol solution in −80°C for long-term storage.

**Table 1:** Geographic origin and NCBI accession number of 63 isolates of *Tilletia horrida* sequenced in this study.

Isolates	State	County/Parish	NCBI accession no.			
			ITS	LSU	*EF1α*	*RPB1*
AR-1	Arkansas	Desha	MZ424381	MZ424318	MZ448515	MZ496315
AR-2	Arkansas	Cross	MZ424382	MZ424319	MZ448516	MZ496316
AR-3	Arkansas	Arkansas	MZ424383	MZ424320	MZ448517	MZ496317
AR-4	Arkansas	Desha	MZ424384	MZ424321	MZ448518	MZ496318
AR-5	Arkansas	Arkansas	MZ424385	MZ424322	MZ448519	MZ496319
AR-6	Arkansas	Cross	MZ424386	MZ424323	MZ448520	MZ496320
AR-7	Arkansas	Arkansas	MZ424387	MZ424324	MZ448521	MZ496321
CA-1	California	Sutter	MZ424388	MZ424325	MZ448522	MZ496322
CA-2	California	Glenn	MZ424389	MZ424326	MZ448523	MZ496323
CA-3	California	Contra Costa	MZ424390	MZ424327	MZ448524	MZ496324
CA-4	California	Contra Costa	MZ424391	MZ424328	MZ448525	MZ496325
CA-5	California	Contra Costa	MZ424392	MZ424329	MZ448526	MZ496326
CA-6	California	Sutter	MZ424393	MZ424330	MZ448527	MZ496327
CA-7	California	Glenn	MZ424394	MZ424331	MZ448528	MZ496328
CA-8	California	Butte	MZ424395	MZ424332	MZ448529	MZ496329
CA-9	California	Butte	MZ424396	MZ424333	MZ448530	MZ496330
CA-10	California	Butte	MZ424397	MZ424334	MZ448531	MZ496331
LA-1	Louisiana	Jefferson Davis	MZ424398	MZ424335	MZ448532	MZ496332
LA-2	Louisiana	Jefferson Davis	MZ424399	MZ424336	MZ448533	MZ496333
LA-3	Louisiana	Acadia	MZ424400	MZ424337	MZ448534	MZ496334
LA-4	Louisiana	Acadia	MZ424401	MZ424338	MZ448535	MZ496335
LA-5	Louisiana	Acadia	MZ424402	MZ424339	MZ448536	MZ496336
LA-6	Louisiana	Jefferson Davis	MZ424403	MZ424340	MZ448537	MZ496337
LA-7	Louisiana	Acadia	MZ424404	MZ424341	MZ448538	MZ496338
LA-8	Louisiana	Acadia	MZ424405	MZ424342	MZ448539	MZ496339
LA-9	Louisiana	Acadia	MZ424406	MZ424343	MZ448540	MZ496340
MO-1	Missouri	Dunklin	MZ424407	MZ424344	MZ448541	MZ496341
MO-2	Missouri	Dunklin	MZ424408	MZ424345	MZ448542	MZ496342
MO-3	Missouri	Dunklin	MZ424436	MZ424373	MZ448570	MZ496370
MS-1	Mississippi	Bolivar	MZ424409	MZ424346	MZ448543	MZ496343
MS-2	Mississippi	Bolivar	MZ424410	MZ424347	MZ448544	MZ496344
MS-3	Mississippi	Bolivar	MZ424411	MZ424348	MZ448545	MZ496345
MS-4	Mississippi	Bolivar	MZ424412	MZ424349	MZ448546	MZ496346
MS-5	Mississippi	Bolivar	MZ424413	MZ424350	MZ448547	MZ496347
MS-6	Mississippi	Bolivar	MZ424414	MZ424351	MZ448548	MZ496348
MS-7	Mississippi	Bolivar	MZ424415	MZ424352	MZ448549	MZ496349
MS-8	Mississippi	Bolivar	MZ424416	MZ424353	MZ448550	MZ496350
MS-9	Mississippi	Bolivar	MZ424417	MZ424354	MZ448551	MZ496351
MS-10	Mississippi	Bolivar	MZ424418	MZ424355	MZ448552	MZ496352
MS-11	Mississippi	Bolivar	MZ424419	MZ424356	MZ448553	MZ496353
MS-12	Mississippi	Bolivar	MZ424420	MZ424357	MZ448554	MZ496354
MS-13	Mississippi	Bolivar	MZ424421	MZ424358	MZ448555	MZ496355
MS-14	Mississippi	Bolivar	MZ424422	MZ424359	MZ448556	MZ496356
MS-15	Mississippi	Bolivar	MZ424423	MZ424360	MZ448557	MZ496357
MS-16	Mississippi	Bolivar	MZ424424	MZ424361	MZ448558	MZ496358
MS-17	Mississippi	Bolivar	MZ424425	MZ424362	MZ448559	MZ496359
MS-18	Mississippi	Bolivar	MZ424426	MZ424363	MZ448560	MZ496360
MS-19	Mississippi	Bolivar	MZ424427	MZ424364	MZ448561	MZ496361
MS-20	Mississippi	Bolivar	MZ424428	MZ424365	MZ448562	MZ496362
MS-21	Mississippi	Bolivar	MZ424429	MZ424366	MZ448563	MZ496363
MS-22	Mississippi	Bolivar	MZ424430	MZ424367	MZ448564	MZ496364
MS-23	Mississippi	Bolivar	MZ424431	MZ424368	MZ448565	MZ496365
MS-24	Mississippi	Bolivar	MZ424432	MZ424369	MZ448566	MZ496366
MS-25	Mississippi	Bolivar	MZ424433	MZ424370	MZ448567	MZ496367
MS-26	Mississippi	Bolivar	MZ424434	MZ424371	MZ448568	MZ496368
MS-27	Mississippi	Bolivar	MZ424435	MZ424372	MZ448569	MZ496369
TX-1	Texas	Jefferson	MZ424437	MZ424374	MZ448571	MZ496371
TX-2	Texas	Jefferson	MZ424438	MZ424375	MZ448572	MZ496372
TX-3	Texas	Jefferson	MZ424439	MZ424376	MZ448573	MZ496373
TX-4	Texas	Jefferson	MZ424440	MZ424377	MZ448574	MZ496374
TX-5	Texas	Chambers	MZ424441	MZ424378	MZ448575	MZ496375
TX-6	Texas	Chambers	MZ424442	MZ424379	MZ448576	MZ496376
TX-7	Texas	Chambers	MZ424443	MZ424380	MZ448577	MZ496377

### DNA Extraction

*Tilletia horrida* isolates growing in PDA plates for 14 days were used for DNA extraction. Mycelium was collected by washing the culture plates with 1% NaCl solution and 100 mg (fresh weight) of the mycelium mass were used for the DNA extraction. DNA was extracted using the fungi/yeast genomic isolation kit (Norgen Biotek Corp., ON, Canada) following the manufacturer’s protocol. The quality of the DNA was checked using the Spectramax quickdrop spectrophotometer (Molecular Devices LLC, San Jose, CA).

### Amplification and DNA Sequencing

Genomic DNA of the *T. horrida* isolates was amplified by PCR using four different genomic regions, consisting of two protein-coding genes: translation elongation factor 1-α (*EF-1α*) and the largest subunit of RNA polymerase II (*RPB1*), and two rRNA regions: ITS1 through 2 regions and D1/D2 domains of the large subunit (LSU) rRNA. The primers used in this study were obtained from previous studies (Fell et al., 2000; Wang et al., 2014) and the conserved primer sequence website of the Vilgalmys Mycology lab-Duke University.^1^ Each PCR reaction mixture was composed of 3 μl of DNA adjusted between 10–50 ng/μl, 12.5 μl of 2x KAPA 2G master mix (KAPA Biosystems, Roche Sequencing, Wilmington, MA, United States), 1.25 μl of forward, 1.25 μl of reverse primers, and 8 μl of water to bring the total reaction volume to 25 μl. PCR parameters for amplifying *EF-1α* and RPB*1* were used in this study were the same as described previously (Wang et al., 2014). Amplification for ITS and LSU were performed as follows: initial denaturation at 94°C for 5 min followed by 40 cycles of denaturation at 30s at 94°C, annealing 15 s at 53.5°C, and elongation at 30s at 72°C; and final elongation at 72°C at 5 min. All PCR amplification was conducted in Biometra TOne Thermocycler (Analytikjena, Jena, Germany). All PCR products were run in 1% agarose gel and visualized in blue light. All PCR products were purified from the electrophoresis gel with Zymoclean Gel DNA recovery kits (Zymoresearch, Irvine, CA, United States) according to manufacturer’s recommendations. The purified PCR products were sequenced using capillary Sanger’s sequencing protocol by external sequencing service provider, Eton Biosciences Inc. (San Diego, CA, United States).

### Phylogeny Constructions

Sequences were manually curated and trimmed for noises at the 5′ and 3′ ends. Consensus sequences from forward and reverse reads were generated by Benchling online.^2^ Sequences were aligned with MAFFT v7.475 (Katoh and Standley, 2013) with accurate alignment method, L-INS-I, built-in MAFFT function of “—adjustdirection” was used to orient the nucleotide sequences in same direction. All sequence alignments were edited and adjusted manually in MEGAX (Kumar et al., 2018). Three different phylogeny analyses were conducted as: Maximum Likelihood (ML; Felsenstein, 1981), Neighbor-Joining (NJ; Saitou and Nei, 1987), and Minimum Evolution (ME; Rzhetsky and Nei, 1993). ML analysis was performed with RaxML version 8.2.12 (Stamatakis, 2014). RaxML analysis was conducted for 1,000 bootstrap replicated with rapid bootstrap analysis with GTRCAT substitution approximation. NJ and ME analyses were performed in R 4.0.3 (R Core Team, 2020) with APE package version 5.4–1(Paradis et al., 2004) using Rstudio (R Studio Team, 2020). NJ analysis was performed in default mode, whereas ME was performed with balanced function (Desper and Gascuel, 2004). Tree topologies were visualized and edited using FigTree v1.4.4 (Rambaut, 2018). Overall, three different sequences sets were used to construct the phylogeny trees.

### Multi-Gene Phylogeny Analyses

Aligned individual sequences were concatenated in different combinations to form three datasets: (1) Ribosomal RNA datasets of 1,075 bp formed by combination of LSU and ITS (including 5.8 rRNA) in that order; (2) Multi-gene datasets of 1,615 bp formed by combination of two protein-coding genes *EF1-α* and *RPB1* in that order; and (3) Multi-gene datasets of 2,690 bp were formed by combining all four sequences, order of genes *EF1-α*, *RPB1*, LSU, and ITS (including 5.8S rRNA). Nucleotide sequence length was approximately 895, 720, 545, and 530 bp for *EF1-α*, *RPB1*, LSU, and ITS (including 5.8S rRNA), respectively. All datasets were subjected to all three phylogeny constructions such as ML, NJ, and ME. *Tilletia horrida* strain QB1 (Bio project no: PRJNA280382; Wang et al., 2015) was used as the reference. *Tilletia controversa* strain DAOMC 236426 (Bio project no: PRJNA393324; Nguyen et al., 2019) was used as an outgroup in the final tree.

### Its Region-Only Phylogeny Analysis

In order to take advantage of the multiple *T. horrida* ITS sequences in the database (with no corresponding protein-coding gene sequences), we performed an ITS region-along sequence analyses. ITS sequences of all 63 *T. horrida* isolates from this study were subjected to the National Center for Biotechnology Institute (NCBI) BLAST (Altschul et al., 1990). All the hits in the NCBI results were downloaded for the analysis. Multiple entries in the result were cross-referenced based on the accession numbers and the duplicates were removed. A total of 172 unique accession numbers of various *Tilletia* spp. were downloaded from NCBI using BioPython 1.78 (Cock et al., 2009) in Python 3.8.5 (Van Rossum and Drake, 2009). Based on the preliminary tree branching pattern, a final ITS region-only phylogeny tree was constructed using 26 *T. horrida* sequences, six *T. barclayana* sequences, and one *T. australiensis* sequence (Table 2). Preliminary ITS region-only phylogeny tree with all 172 *Tilletia* spp. isolates, NCBI accession numbers, and other information are available in supplementary (Supplementary Table S1; Figure S2).

**Table 2:** Primers of ITS, LSU, *EF-1α,* and *RPB1* used in this study.

Name	Locus	Primers (5′-3′)	*Tm*^a^ (Cock et al., 2009)	References
Internal transcriber space	ITS	ITS1: TCC GTA GGT GAA CCT GCG G ITS4: TCC TCC GCT TAT TGA TAT GC	59.5 52.1	Fell et al., 2000
D1/D2 domains of the large subunit of rRNA	LSU	F63: GCA TAT CAA TAA GCG GAG GAA AAG LR3: GGT CCG TGT TTC AAG ACG G	54.2 56.2	
Elongation Factor	*EF-1α*	EF1-983F: GCY CCY GGH CAY CGT GAY TTY AT EF1-2218R: ATG ACA CCR ACR GCR ACR GTY TG	61.2 60.9	Wang et al., 2014
largest subunit of RNA polymerase II	*RPB1*	RPB1-Af: GAR TGY CCD GGD CAY TTY GG RPB1-Cr: CCN GCD ATN TCR TTR TCC ATR TA	57.8 54.2	

a Melting temperature of the primer.

### Analysis of Diversity and Recombination Rates

DnaSP v6.0 (Rozas et al., 2017) was used to determine nucleotide diversity and the minimum number of recombination events. Similarly, DnaSP v 6.0 (Rozas et al., 2017) was also used for the calculation of class I neutrality tests: Tajima’s D and Fu and Li’s D* and F*, for detecting departure from the mutation/drift equilibrium (Tajima, 1989; Fu and Li, 1993). For the above-mentioned calculation, only *T. horrida* isolates were considered in multi-gene sequence sets and *T. controversa* was used as an outgroup as needed. However, for ITS region-only sequence sets, *T. barclayana* strain 104 was used as an outgroup as needed.

### Nucleotide Sequence Accession Numbers

All the sequenced genes have been deposited into the National Center for Biotechnology Institute (NCBI) database under the following accession numbers: LSU, MZ424318–MZ424380, ITS, MZ424381–MZ424443, *EF1*, MZ448515–MZ448577, and *RPB1*, MZ496315–MZ496377.

## Results

### Its Region-Only Phylogeny Characterization

**Figure 2:**
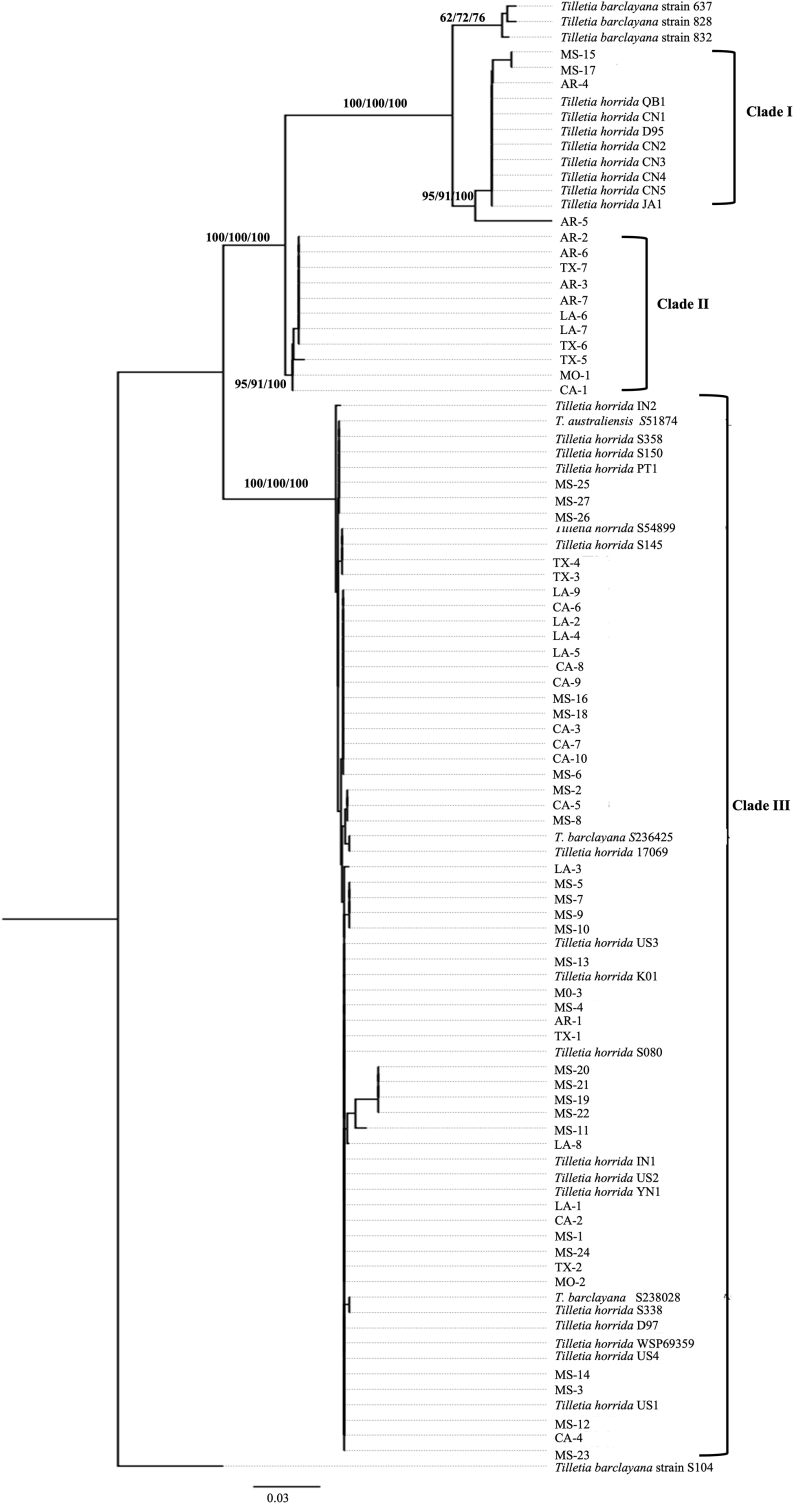
ITS region-based neighbor-joining phylogenetic tree with 63 *T. horrida* isolates collected Arkansas (AR), California (CA), Louisiana (LA), Mississippi (MS), Missouri (MO), and Texas (TX) in the US in the current study, along with 33 ITS sequences obtained from the NCBI database. *T. horrida* strain QB1 was used as a reference isolate and *T. barclayana* S104 was used as an outgroup. The scale bar represents the number of substitutions per site. The values on the branches indicate the percentage of trees based on 1,000 bootstrap replicates on ML/NJ/ME, respectively. Only branches values with >50% replicates are shown.

We took advantage of the ITS region sequences available on multiple isolates in the databases and conducted phylogenetic analysis first based on the ITS region-only. For the analysis, we downloaded 172 various *Tilletia* spp. from NCBI and constructed a phylogenetic tree. Most of the isolates formed species-specific clades (Supplementary Figure S2). Hence, the final tree was constructed with 63 *T. horrida* isolates collected from this study, along with a subset of the sequences from the NCBI database, including 26 *T. horrida* isolates, six *T. barclayana* isolates, and one *T. australiensis* isolate downloaded from the NCBI database. All *T. barclayana* isolates were included in the final tree due to taxonomy controversy of the kernel smut fungus. Phylogeny based on ITS region (Figure 2) shows three different groups of the isolates. Most isolates (76%) were clustered together along with 22 isolates from eight different countries (Australia, China, India, Korea, Pakistan, Taiwan, the US, and Vietnam) in Clade III (Figure 2; Table 3). Out of the remaining 15 isolates, 11 *T. horrida* isolates collected in this study were clustered together in clade II. All the isolates clustered in clade II were from the current study. Clade I was grouped by clustering of seven isolates from China, one from Japan, and four *T. horrida* isolates from the current study (Figure 2; Table 3).

**Table 3:** NCBI accession number, host, and country of origin of the strains of *Tilletia australiensis, T. barclayana*, and *T. horrida* used in the final ITS-only region phylogenetic analysis.

Name of Isolate	Strain name	NCBI accession no.	Host	Country of origin	References
*T. australiensis*	BRIP 51874	MH231774.1	*Oryza rufipogon*	Australia	McTaggart and Shivas, 2018
*T. barclayana*	S637	AF310170.1	*Paspalum distichum*	United States	Levy et al., 2001
*T. barclayana*	S828	AF310169.1	*Paspalum obtusum*	United States	Levy et al., 2001
*T. barclayana*	S832	AF310168.1	*Paspalum distichum*	United States	Levy et al., 2001
*T. barclayana*	DAOM236425	HQ317521.1	*Oryza sativa*	United States	Liu et al., 2014
*T. barclayana*	DAOM238028	HQ317541.1	*Oryza sativa*	United States	Liu et al., 2014
*T. barclayana*	104	AF399894.1	*Pennisetum orientale*	China	Zhang et al., 2001
*T. horrida*	T 54899	MH231786.1	*Oryza sativa*	Australia	McTaggart and Shivas, 2018
*T. horrida*	QB-1	LAXH01000427.1	Rice	China	Wang et al., 2015
*T. horrida*	CN1	DQ827699.1	*Oryza sativa*	China	Zhou et al., 2006
*T. horrida*	CN2	DQ827700.1	*Oryza sativa*	China	Zhou et al., 2006
*T. horrida*	CN3	DQ827701.1	*Oryza sativa*	China	Zhou et al., 2006
*T. horrida*	CN4	DQ827702.1	*Oryza sativa*	China	Zhou et al., 2006
*T. horrida*	CN5	DQ827703.1	*Oryza sativa*	China	Zhou et al., 2006
*T. horrida*	D95	DQ827704.1	*Oryza sativa*	China	Zhou et al., 2006
*T. horrida*	D97	DQ827705.1	*Oryza sativa*	China	Zhou et al., 2006
*T. horrida*	S080	AF398435.1	*Oryza sativa*	China	Zhang et al., 2001
*T. horrida*	S145	AF399892.1	*Oryza sativa*	China	Zhang et al., 2001
*T. horrida*	S150	AF399893.1	*Oryza sativa*	China	Zhang et al., 2001
*T. horrida*	IN1	DQ827706.1	–^a^	India	Zhou et al., 2006
*T. horrida*	Isolate 2	AY560653.2	–	India	Thirumalaisamy et al., 2007b
*T. horrida*	RB1	AY425727.2	–	India	Thirumalaisamy et al., 2007a
*T. horrida*	JA1	DQ827707.1	*Oryza sativa*	Japan	Zhou et al., 2006
*T. horrida*	K01	DQ827714.1	*Oryza sativa*	South Korea	Zhou et al., 2006
*T. horrida*	17,069	LC494385.1	*Oryza sativa*	Taiwan	Ou and Chen, 2019
*T. horrida*	PT1	DQ827708.1	*Oryza sativa*	Pakistan	Zhou et al., 2006
*T. horrida*	US1	DQ827709.1	*Oryza sativa*	United States	Zhou et al., 2006
*T. horrida*	US2	DQ827710.1	*Oryza sativa*	United States	Zhou et al., 2006
*T. horrida*	US3	DQ827711.1	–	United States	Zhou et al., 2006
*T. horrida*	US4	DQ827712.1	*–*	United States	Zhou et al., 2006
*T. horrida*	338	AF310172.1	*Oryza sativa*	United States	Levy et al., 2001
*T. horrida*	358	AF310173.1	*Oryza sativa*	United States	Levy et al., 2001
*T. horrida*	WSP69539	AF310171.1	*Oryza sativa*	United States	Levy et al., 2001
*T. horrida*	YN1	DQ827713.1	*Oryza sativa*	Vietnam	Zhou et al., 2006

a Host information was not provided in the database.

### Multi-Gene Phylogeny Characterization

**Figure 3:**
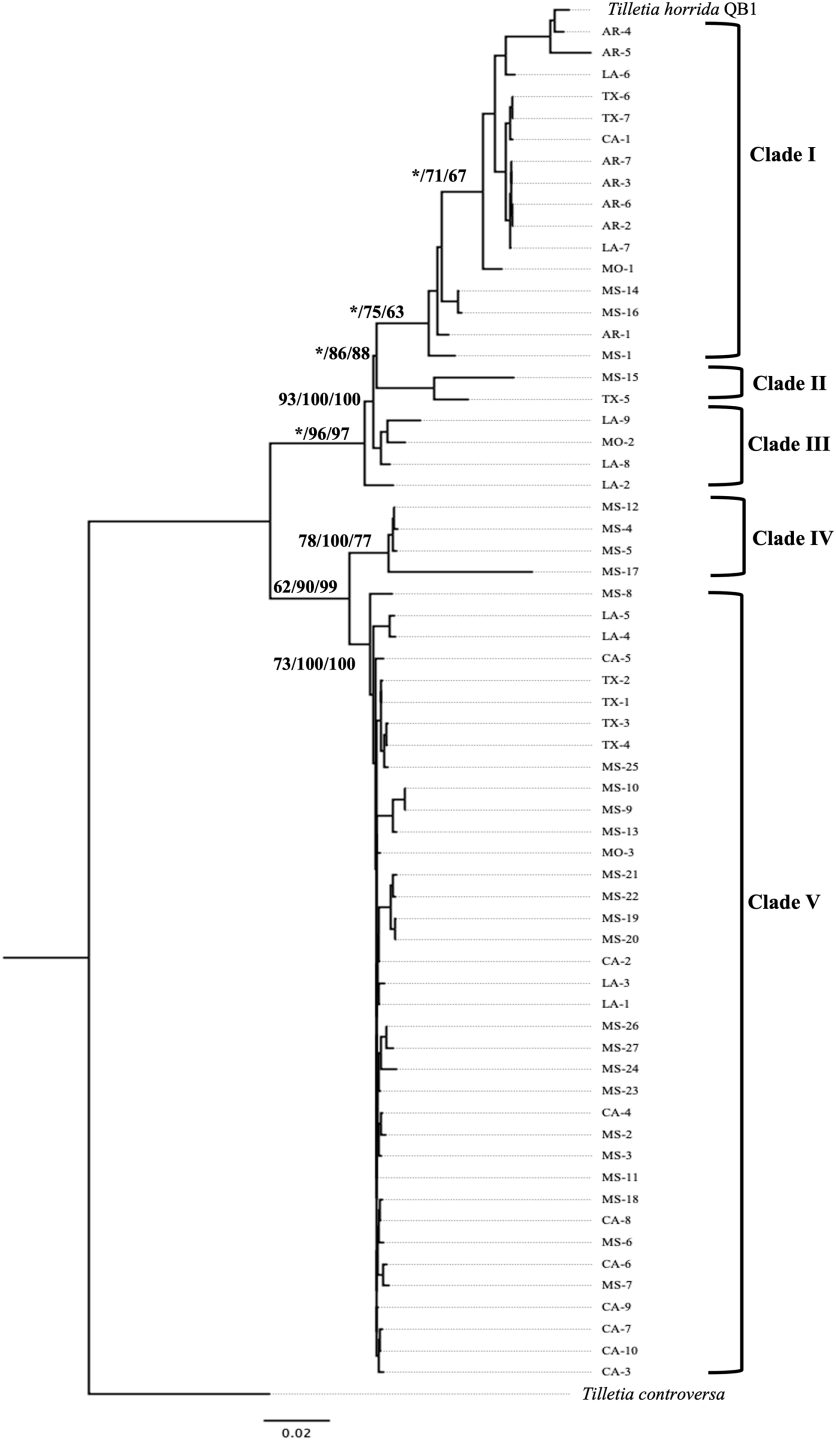
Neighbor-joining phylogenetic tree of concatenated sequences with EF*-1α, RPB1*, LSU, and ITS of 63 isolates collected from Arkansas (AR), California (CA), Louisiana (LA), Mississippi (MS), Missouri (MO), and Texas (TX) in the US. *T. horrida* strain QB1 was used as a reference isolate and *T. controversa* strain DAOMC 236426 was used as an outgroup. The scale bar represents the number of substitutions per site. The value on the branches indicates the percentage of trees based on 1,000 bootstrap replicates on ML/NJ/ME, respectively. Only branches values with >50% replicates are show; “*” indicates that the branch value is less than 50%.

The concatenated sequence *EF1α-RPB1*-LSU-ITS of 63 *T. horrida* isolates with 2,960 bp nucleotide was aligned and a phylogenetic tree was calculated with three different phylogenetic analyses. Phylogenetic analyses clustered the 63 isolates into five different groups (Figure 3). Most isolates (59%) were grouped together in clade V, which consists of the isolates collected from five different states (California, Louisiana, Mississippi, Missouri, and Texas). Most isolates collected from Mississippi (19/27), California (9/10), and Texas (4/7) were clustered in clade V. The isolates collected from Louisiana (4/9) and Missouri (1/3) were also grouped in clade V (Figure 3; Table 1).

Similarly, 27% of the 63 *T. horrida* isolates were clustered in clade I along with the reference isolate *T. horrida* strain QB1. All *T. horrida* isolates collected from Arkansas (7/7) were grouped in clade I, along with a few isolates collected from California (1/9), Louisiana (2/9), Missouri (1/3), Mississippi (3/27), and Texas (2/7). Additionally, clade III and clade IV clustered with four isolates each from Mississippi and Louisiana. Clade III consisted of the isolates collected from Louisiana (3/9) and Missouri (1/3), whereas all the isolates grouped in clade IV were collected from Mississippi (4/27). Two isolates, MS-15 and TX-5, were the only isolates to be grouped in clade II (Figure 3; Table 1).

### rRNA Regions-Based Phylogeny Characterization

**Figure 4:**
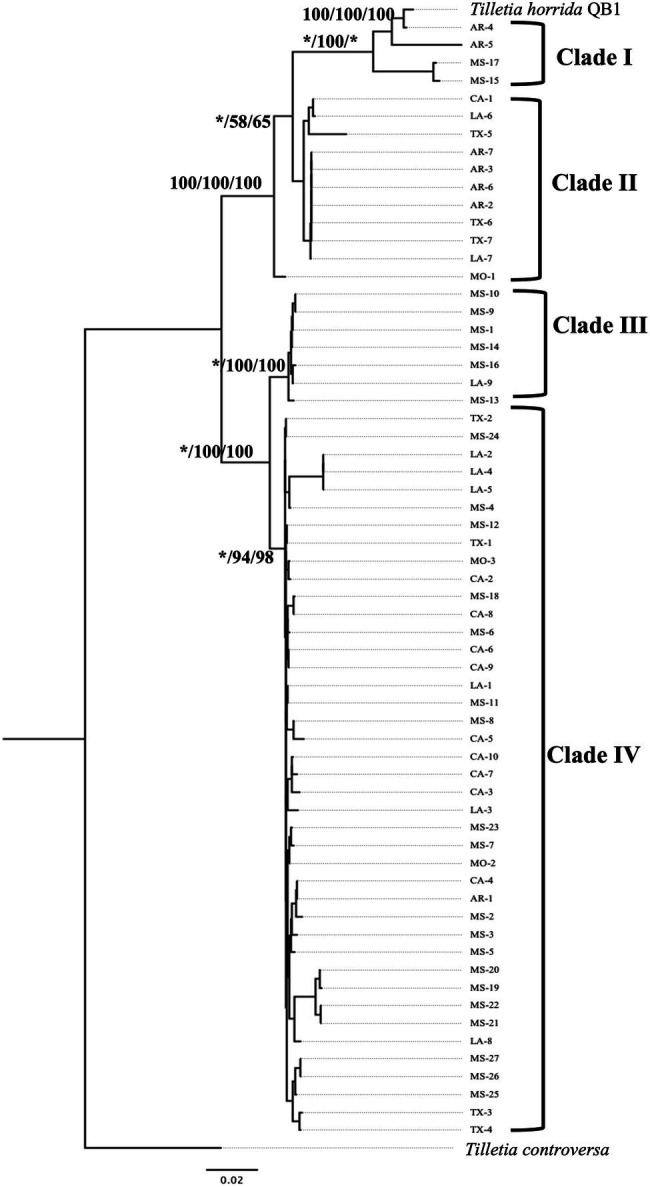
Neighbor-joining phylogenetic tree of concatenated sequences with rRNA regions LSU, and ITS of 63 isolates collected from Arkansas (AR), California (CA), Louisiana (LA), Mississippi (MS), Missouri (MO), and Texas (TX) in the US. *T. horrida* strain QB1 was used as a reference isolate and *T. controversa* strain DAOMC 236426 was used as an outgroup. The scale bar represents the number of substitutions per site. The value on the branches indicates the percentage of trees based on 1,000 bootstrap replicates on ML/NJ/ME, respectively. Only branches values with >50% replicates are shown; “*” indicates that the branch value is less than 50%.

Multi-locus phylogeny of the concatenated of LSU-ITS (including 5.8S) sequences of 63 isolates of T. *horrida* with 1,065 bp nucleotide was aligned and phylogenetic analyses were conducted. Our rRNA regions-based phylogenetic analysis clustered the 63 *T. horrida* isolates in four clades (Figure 4). Most of the isolates (76%) were grouped together in clade IV. Most isolates collected from Mississippi (20/27), California (9/10), Louisiana (7/9), and Texas (4/7), along with the isolates collected from Arkansas (1/7) and Missouri (2/3) were clustered together in clade IV (Figure 4; Table 1).

Similarly, two isolates from Arkansas and Mississippi each were grouped together in clade I, along with the reference isolates *T. horrida* strain QB1. Clade II consisted of the isolates collected from Arkansas (4/7), California (1/10), Louisiana (2/9), and Texas (3/7). Similarly, clade III consisted of five isolates from Mississippi and one isolate from Louisiana. Isolate MO-1 did not group with clades and monophyletically branched with clade I and clade II (Figure 4; Table 1).

### Protein-Coding Gene-Based Phylogeny Characterization

**Figure 5:**
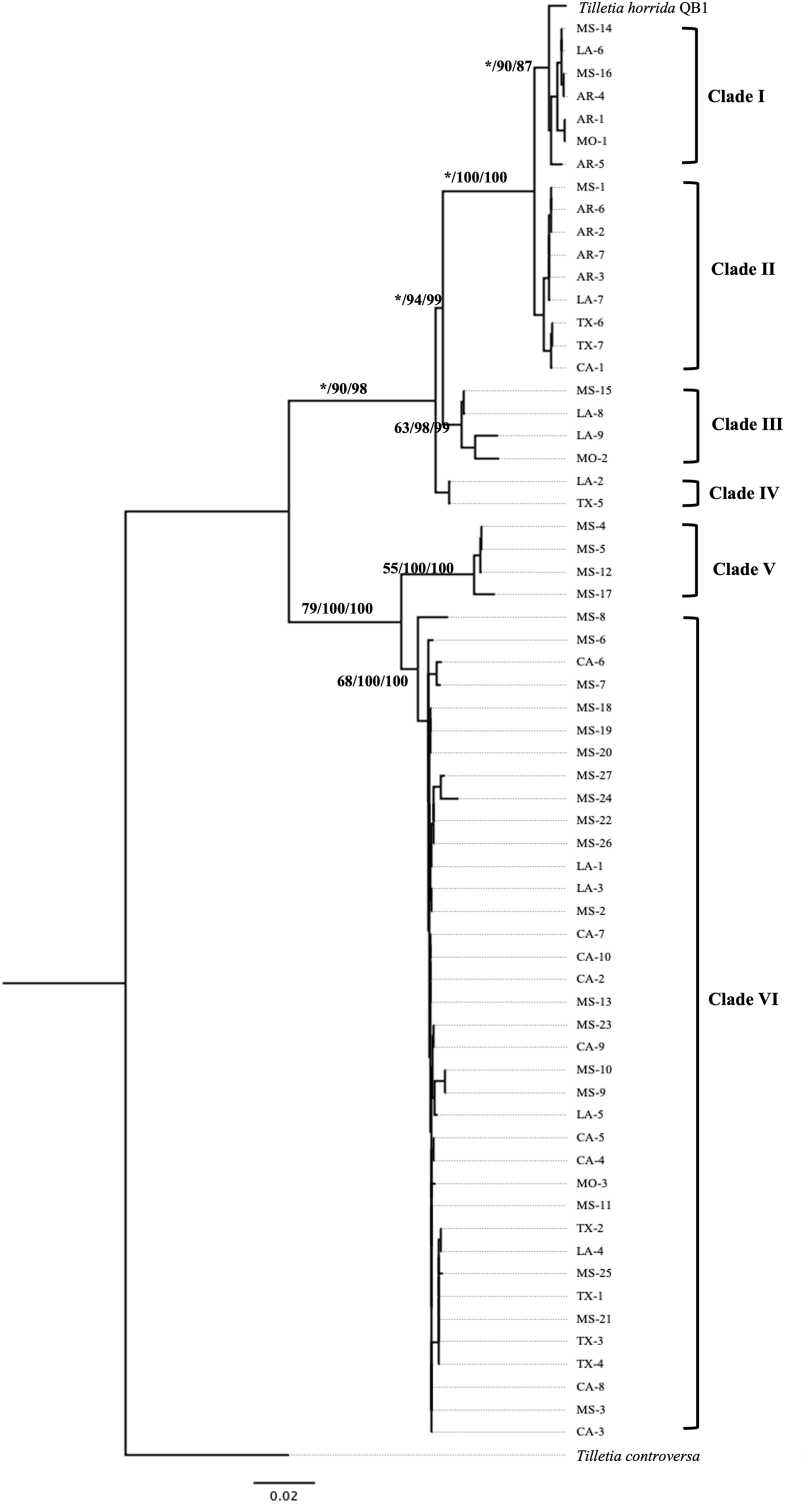
Neighbor-joining phylogenetic tree of concatenated sequences with protein coding genes EF*-1α* and *RPB1*of 63 isolates collected from Arkansas (AR), California (CA), Louisiana (LA), Mississippi (MS), Missouri (MO), and Texas (TX) in the US. *T. horrida* strain QB1 was used as a reference isolate and *T. controversa* strain DAOMC 236426 was used as an outgroup. The scale bar represents the number of substitutions per site. The value on the branches indicates the percentage of trees based on 1,000 bootstrap replicates on ML/NJ/ME, respectively. Only branches values with >50% replicates are show; “*” indicates that the branch value is less than 50%.

Multi-locus phylogeny of the concatenated of *EF1-α* and *RPB1* sequences of 63 isolates of *T. horrida* with 1,615 bp nucleotide was aligned and phylogenetic analyses were conducted. Our protein-coding gene-based phylogenetic analysis clustered the 63 *T. horrida* isolates in six clades (Figure 5). Most of the isolates (60%) were grouped together in clade VI. Most isolates collected from Mississippi (19/27), California (9/10), and Texas (4/7), along with isolates collected from Louisiana (4/9) and Missouri (1/3) were clustered together in clade VI (Figure 5; Table 1).

Similarly, clade I consisted of three isolates collected from Arkansas (3/7) and one isolate each from Mississippi, Missouri, and Louisiana. Similarly, clade II consisted of four isolates collected from Arkansas, and one isolate each from California, Mississippi, and Louisiana, along with two isolates from Texas. Similarly, clade III consisted of two isolates collected from Louisiana and isolate each from Missouri and Mississippi. Clade IV consisted of only two isolates, one from Louisiana and the other from Texas, whereas clade V were clustered with four isolates all collected from Mississippi.

### Analysis of Diversity and Recombination Rates

Diversity parameters and neutrality test were calculated with concatenated sequence of all four regions and individually by DnaSP 6.0 (Rozas et al., 2017). Test of neutrality showed non-significant drift from mutation equilibrium in both Tajima’s D and Fu and Li’s D* and F* statistics for both ITS-only region and multi-locus concatenated sequence. Number of recombination event was predicted to be 4 and 50 in ITS-only sequence and multi-locus sequence sets, respectively (Table 4.)

**Table 4:** Sequence variation statistics of the kernel smut fungal populations in the US^a^.

Sequence set	Diversity parameters^b^					Neutrality test			
	*n*	*S*	ND	θ*_w_*	NM	Tajima’s D	Fu and Li’s D*	Fu and Li’s F*	R^c^
ITS-only^d^	91	96	0.05515	18.88	139	0.43 (NS)^e^	0.588 (NS)	0.62 (NS)	4
Multi-gene	64	427	0.053	90.308	469	1.49 (NS)	1.61(NS)	1.82(NS)	50

a All calculations were made using DnaSP v6.0 software.

b n, number of strains; S, total number of segregating sites; ND, Nucleotide diversity; θ_w_ = Watterson’s theta; and NM, number of mutations.

c number of recombination events.

d While calculating various parameter in the ITS-only sequence sets, four T. barclayana sequences which formed outgroups were removed from the calculation.

e NS, not significant.

Among the individual regions nucleotide diversity (ND), Watterson’s theta (θ_w_) and segregating sites were found to be highest in *RPB1* region as compared to the other three regions. Test of neutrality showed significant drift from mutation equilibrium in both Tajima’s D and Fu and Li’s D* and F* statistics in *RPB1* region, whereas only Fu and Li D* and F* statistics were significant in rRNA regions and *EF1α* region (Supplementary Table S2).

## Discussion

Kernel smut of rice has emerged as one of the most important diseases, threatening the US rice production. Economic impact of kernel smut is more significant from the loss of quality than from the loss of yield. In recent years, an increase in the severity and incidence of kernel smut and in cases of rejection of rice at selling point has been reported across the US, especially in Texas. In this study, we investigated the genetic diversity of the *T. horrida* populations in the US. Genomic DNA was extracted from the 63 isolates of *T. horrida* collected from Arkansas, California, Louisiana, Missouri, Mississippi, and Texas and subjected to multi-locus sequence analysis (MLSA). The results of our study showed the presence of genetically diverse *T. horrida* populations in the US. To our knowledge, this is the first study to analyze multi-locus region of the *T. horrida* populations in rice.

Our research reveals that there were five groups of the *T. horrida* populations in the US. *Tilletia horrida* clusters did not correspond with the geographical origin of the isolates collected. Only the isolates collected from Arkansas were clustered together in only one clade (clade I) with the reference isolate *T. horrida* strain QB1. Along with the Arkansas population, the isolates from California were the least diverse, whereas the population in Louisiana and Mississippi were most diverse as they grouped together in 3 out of 5 clades. Low diversity within the California population found in the current study can be attributed to quarantine practice that has been enforced in the state to prevent the introduction of disease, insect, and weed pests in rice seed into California from the southern region of the US and from foreign countries for many years (Oscar, 2006). The results of our research here are in contrast with those of *Ustilaginoidea virens*, the causal agent of false smut of rice, where a higher level of genetic differentiation among the *U. virens* populations found in the study with the isolates collected from geographically distant rice-growing areas of China (Sun et al., 2013). Unlike another bunt pathogen *T. indica* (Singh and Gogoi, 2011), the lack of correlation between genetic diversity and geographical specificity among the *T. horrida* populations in the current study may be attributed to unrestricted trading of rice seeds and lack of quarantine restriction in the southern US. In addition, the current study indicates that there are some levels of genetic differentiation in the *T. horrida* populations. These results have a direct implementation on the development of new rice cultivars with improved resistance to kernel smut. Future efforts toward breeding for resistant cultivars against kernel smut should consider selecting representative isolates from each of genetically diverse groups in the process of kernel smut resistance screening.

Neutrality tests suggest there was no significant departure from the mutation drift equilibrium, indicating the *T. horrida* population does not deviate from natural expectation in Tajima’s D and Fu and Li’s D* and F* tests (Tajima, 1989; Fu and Li, 1993). Individual phylogenetic analyses of all four regions showed higher nucleotide substitution in *RPB1* and ITS as compared to the *EF1⋅* and LSU regions. Along with higher nucleotide substitution per site, *RPB1* also had highest nucleotide diversity (ND), Watterson’s theta (θ_w_), and segregating sites. Similarly, based on Tajima’s D test, only *RPB1* deviates from the mutation equilibrium with significant and positive Tajima’s D. Variation statistics showed 50 recombination events among the concatenated sequence. This might be due to the possible sexual recombination between different isolates. *Tilleita horrida* is a hemi-biotrophic fungus, which probably facilitates such recombination through mating of compatible types. However, no clear evidence of compatible sexual mating is still unknown in *Tilletia horrida* (Carris et al., 2006) or in similar non-systematic bunt *Tilletia indica* (Goates, 1988; Gupta et al., 2015).

Along with multi-locus analysis of the *T. horrida* populations in the US, we also analyzed 33 other *Tilletia* spp., including *T. horrida* and *T. barclayana* reported from eight different countries. Due to the lack of global information on multi-locus regions on the *T. horrida* isolates, we analyzed ITS regions-only reported in the NCBI database. Our analysis revealed that there are three groups of *T. horrida* isolates distributed in the world. The majority (76%) of the *T. horrida* isolates from this study, along with six other isolates reported previously from the US (Levy et al., 2001), was clustered together along with 15 other smut isolates from eight different countries. Similarly, four *T. horrida* isolates from this study were clustered together with seven *T. horrida* isolates reported from China (Zhou et al., 2006; Wang et al., 2015). Kernel smut is seedborne and rice is one of the most traded crops around the world (USDA, 2020). Our ITS regions-only analysis suggests potential multiple entries of the kernel smut pathogen from foreign countries into the US.

In the current study, clade III also clustered with *T. australiensis* isolated from wild rice (*O. rufipogon*) in Australia (McTaggart and Shivas, 2018) in addition to *T. horrida* isolates isolated from rice. ITS region sequence showed 99.75% similarity between *T. australiensis* and *T. horrida* strain 54,899 isolated from rice in Australia. Such high percentage of similarity between the kernel smut isolates from wild and cultivated rice indicates that the kernel smut fungus can infect multiple hosts and that wild rice potentially serves as an alternative host to the pathogen. The results of previously unverified reports indicate that *T. horrida* may infect *Digitaria* Haller, *Leeria* Sw., and *Panicum* L. (Tracy and Earle, 1896) and *Pennisetum* L. C. (Tullis and Johnson, 1952). However, until this date, there is no clear evidence that the *T. horrida* fungus can infect multiple hosts other than rice under natural conditions (Carris et al., 2006). Out of six *T. barclayana* isolates only two strains, DAOM 236425 and DAOM 238028, clustered together in clade III. Two *T. barclayana* strains clustered in clade III were the only *T. barclayana* isolated from rice (Liu, 2014), whereas other *T. barclayana* strains were isolated either from *Paspalum* spp. or *Pennisetum orientale*. Other strains formed a separate group from *T. horrida* isolates. Kernel smut isolated from other grasses clearly form different branches in the phylogenetic tree. Difference in the lineage of *T. horrida* and *T. barclayana* isolates have also been demonstrated in previous studies (Levy et al., 2001; Castlebury et al., 2005).

In conclusion, we can parse finger genetic diversity in the kernel smut fungus, *T. horrida*, using multi-locus sequence analysis. This is the first study analyzing the genetic diversity among the *T. horrida* populations in the US. Our study demonstrates the presence of genetically diverse of *T. horrida* isolates in different rice-growing states. Higher than 99% similarity in ITS region sequences between *T. horrida* isolates and between different countries may be attributed to the wide distribution of smutted rice along with global trade. The understanding of the genetic diversity of the *T. horrida* populations from the current study will help researchers develop effective host resistance and chemical management strategies, especially cultivar resistance, for the control of kernel smut of rice.

## Data Availability Statement

The datasets presented in this study can be found in online repositories. The names of the repository/repositories and accession number(s) can be found in the article/Supplementary Material.

## Author Contributions

SK, SA-B, SG, and X-GZ conceived and designed the experiments. SK and SG performed the isolations of the fungus and genomic DNA extractions. SK performed all sequencing experiments and wrote the manuscript. SK and SA-B analyzed the data. All authors have read and approved the manuscript.

## Funding

This work was supported, in part, by USDA NIFA OREI (2015-51300-24286) and Texas Rice Research Foundation (TRRF 2018–2021).

## Conflict of Interest

The authors declare that the research was conducted in the absence of any commercial or financial relationships that could be construed as a potential conflict of interest.

## Publisher’s Note

All claims expressed in this article are solely those of the authors and do not necessarily represent those of their affiliated organizations, or those of the publisher, the editors and the reviewers. Any product that may be evaluated in this article, or claim that may be made by its manufacturer, is not guaranteed or endorsed by the publisher.

## Footnotes

^1^ https://sites.duke.edu/vilgalyslab/rdna_primers_for_fungi/

^2^ www.benchling.com
