# Supplementary material for: Multi-Locus Sequence Analysis Reveals Diversity of the Rice Kernel Smut Populations in the United States
Source: Front Microbiol. 2022 May 4;13:874120. doi: 10.3389/fmicb.2022.874120 (PMC9116506; doi:10.3389/fmicb.2022.874120)
Supplement: Supplementary file 1 [file Data_Sheet_1.docx]

**Supplementary Figures and Tables**

**SUPPLEMENTARY FIGURE S1.** Phylogenetic trees using four *T. horrida* fungal genes *EF-1α, RPB1*, LSU, and ITS, *Tilletia horrida* strain QB1 was used as a reference isolate and *T. controversa* strain DAOMC 236426 was used as an outgroup. The scale bar represents the number of substitutions per site. The value on the branches indicates the percentage of trees based on 1000 bootstrap replicates on ML/NJ/ME, respectively. Only the values with >50% replicates are shown; “*” indicates that the branch value is less than 50%.

**SUPPLEMANTARY FIGURE S2.** Phylogenetic trees calculated using ITS region of 172 *Tilletia* spp. downloaded from the NCBI database in comparison with 63 *T. horrida* isolates in this study. The scale bar represents the number of substitutions per site. The value on the branches indicates the percentage of trees based on 1000 bootstrap replicates on ML/NJ/ME, respectively. Only the values with >50% replicates are shown. The description of the isolates used in this study is available in the supplementary Table S1.

**SUPPLEMENTARY TABLE S1.** A list of species, strain, NCBI accession number, with country of origin, and host of the isolates of various *Tilletia* species described in SUPPLEMENTARY FIGURE S1.

| **Clade** | **S. N** | **Species name** | **Strain name** | **NCBI accession** | **Country of origin** | **Host** |
| --- | --- | --- | --- | --- | --- | --- |
| Various *Tilletia* sp. Clade | 1 | *T. barclayana* | Strain 637 | AF310170.1 | USA | *Paspalum distichum* |
|  | 2 | *T. barclayana* | Strain 832 | AF310168.1 | USA | *Paspalum distichum* |
|  | 3 | *T. barclayana* | Strain 828 | AF310169.1 | USA | *Panicum obtusum* |
|  | 4 | *T. cape-yorkensis* | Voucher BRIP 27011 | MH231778.1 | Australia | *Whitechloa airoides* |
|  | 5 | *T. chionachnes* | Voucher BRIP 26898 | MH231779.1 | Australia | *Chionachne cyathopoda* |
|  | 6 | *T. eragrostiellae* | Voucher HUV 15805 | MH231782.1 | India | *Eragrostiella bifaria* |
|  | 7 | *T. filisora* | Voucher BRIP 47729 | MH231784.1 | Thailand | *Cenchrus polystachios* |
|  | 8 | *T. geeringii* | BRIP 51851 (ITS from TYPE material) | NR_156554.1 | Australia | *Eriachne festucacea* |
|  | 9 | *T. geeringii* | Voucher BRIP 51851 | KF055226.1 | Australia | *Eriachne festucacea* |
|  | 10 | *T. gigacellularis* | Voucher HUV 20555 | MH231785.1 | Mexico | *Bouteloua repens* |
|  | 11 | *T. iowensis* | AFTOL-ID 1712 | DQ832253.1 | China | *Phragmites communis* |
|  | 12 | *T. iowensis* | Voucher HUV 17453 | MH231788.1 | India | *Ischaemum rugosum* |
|  | 13 | *T. ischaemi* | Voucher HUV 17453 | NR_156555.1 | Australia | *Eriachne burkittii* |
|  | 14 | *T. kimberleyensis* | Voucher BRIP 51857 | MH231789.1 | Australia | *Chionachne cyathopoda* |
|  | 15 | *T. lageniformis* | Voucher BRIP 47749 | MH231791.1 | Thailand | *Hyparrhenia rufa* |
|  | 16 | *T. lineata* | Voucher BRIP 26844 | MH231793.1 | Australia | *Whitechloa* sp. |
|  | 17 | *T. mactaggartii* | BRIP 51853(ITS type material) | NR_156555.1 | Australia | *Eriachne burkittii* |
|  | 18 | *T. mactaggartii* | Voucher BRIP 51583 | KF055227.1 | Australia | *Eriachne burkittii* |
|  | 19 | *T. mactaggartii* |  | KF055228.1 | Australia | *Eriachne burkittii* |
|  | 20 | *T. marjaniae* | Voucher BRIP 49721 | KF055224.1 | Australia | *Eriachne pulchella* subsp. *dominii* |
|  | 21 | *T. micrairae* | BRIP 52433 | FJ862995.1 | Australia | - |
|  | 22 | *T. moliniae* | Voucher GLM F063830 | KY424489.1 | Germany | *Molinia caerulea* |
|  | 23 | *T. moliniae* | Voucher HUV 12297 | EU659129.1 | Italy | *Phragmites australis* (Cav.) Trin. Ex Steud |
|  | 24 | *T. moliniae* | Voucher HUV 21147 | EU659133.1 | Germany | *Phragmites australis* (Cav.) Trin. Ex Steud |
|  | 25 | *T. moliniae* | Voucher TUB 018197 | EU659136.1 | Germany | *Molinia caerulea* (L.) Moench |
|  | 26 | *T. moliniae* | Voucher TUB 018198 | EU659127.1 | Germany | *Molinia caerulea* (L.) Moench |
|  | 27 | *T. moliniae* | Voucher TUB 018200 | EU659126.1 | Germany | *Molinia caerulea* (L.) Moench |
|  | 28 | *T. moliniae* | Voucher TUB 018201 | EU659131.1 | Germany | *Phragmites australis* (Cav.) Trin. Ex Steud |
|  | 29 | *T. moliniae* | Voucher TUB 018203 | EU659132.1 | Germany | *Phragmites australis* (Cav.) Trin. Ex Steud |
|  | 30 | *T. moliniae* | Voucher TUB 018204 | EU659137.1 | Germany | *Phragmites australis* (Cav.) Trin. Ex Steud |
|  | 31 | *T. moliniae* | Voucher TUB 018922 | EU659134.1 | Poland | *Phragmites australis* (Cav.) Trin. Ex Steud |
|  | 32 | *T. moliniae* | Strain USGS21 | MT000594.1 | Ohio, USA | *Phragmites australis* subsp. *americanus* |
|  | 33 | *T. opaca* | Strain 017 | AF399884.1 | China | *Spinifex longifolius* |
|  | 34 | *T. opaca* | Voucher BRIP 27896 | MH231798.1 | Australia | *Spinifex longifolius* |
|  | 35 | *T. panici-humilis* | Voucher HUV 205832 | MH231799.1 | India | *Panicum humile* |
|  | 36 | *T. pseudochaetochloae* | Voucher BRIP 46730 | MH231800.1 | Australia | *Pseudochaetochloa australiensis* |
|  | 37 | *T. pseudoraphidis* | Voucher BRIP 51873 | MH231801.1 | Australia | *Pseudoraphis spinescens* |
|  | 38 | *T. pulcherrima* |  | EU915293.1 | Texas, USA | *Panicum virgatum* |
|  | 39 | *T. rugispora* | Voucher BRIP 47127 | MH231803.1 | Australia | *Paspalum plicatulum* |
|  | 40 | *T. savilei* | Strain 097 | AF399885.1 | China | *Tripogon jacquemontii* |
|  | 41 | *T. sehimicola* | Voucher BRIP 51847 | MH231804.1 | Australia | *Sehima nervosum* |
|  | 42 | *T. setariae-pumilae* | Voucher HUV 21399 | MH231806.1 | India | *Setaria pumila* |
|  | 43 | *T. setariae-parviflorae* | Voucher BRIP 47735 | MH231805.1 | Thailand | *Setaria parviflora* |
|  | 44 | *T. shivasii* | Voucher BRIP 52525 | MH231807.1 | Australia | *Arundinella nepalensis* |
|  | 45 | *T. sporoboli* | Voucher HUV 1880 | MH231808.1 | Zimbabwe | *Sporobolus festivus* |
|  | 46 | *T. sumatii* | Voucher HUV 17529 | MH231809.1 | India | *Coix lacryma-jobi* |
|  | 47 | *T. sumatii* | Strain 056 | AF399886.1 | China | *Coix lacryma-jobi* |
|  | 48 | *T. tarchypogonis* | Voucher HUV 19626 | MH231812.1 | Mexico | *Trachypogon secundus* |
|  | 49 | *T. tarchypogonis* | Voucher KRAM F-57398 | KX622646.1 | Africa | *Trachypogon spicatus* |
|  | 50 | *T. tarchypogonis* | Voucher KRAM F-57400 | KX622645.1 | Africa | *Trachypogon spicatus* |
|  | 51 | *T. whiteochloae* | Voucher BRIP 51838 | MH231815.1 | Australia | *Whitechloa sp.* |
|  | 52 | *T. xerochloae* | Voucher BRIP 54437 | MH231816.1 | Australia | *Xerochloa imberbis* |
|  | 53 | *Tilletia sp.* | Vega 377 | EU009972.1 | Colombia | *Coffea arabica* |
|  | 54 | *Tilletia sp.* | Vega 383 | EU009971.1 | Colombia | *Coffea arabica* |
|  | 55 | Uncultured fungus clone | CMH52 | KF800671.1 | Missouri, USA | indoor air |
|  | 56 | Uncultured fungus clone | FITS URP2 W56 | HQ436099.1 | Singapore | *Axonopus compressus* soil |
| *Tilletia maclaganii* Clade | 1 | *T. maclaganii* | PKLE-1 | MH397305.1 | Texas, USA | *Panicum virgatum* |
|  | 2 | *T. maclaganii* | PKLE-2 | MH397304.1 | Texas, USA | *Panicum virgatum* |
|  | 3 | *T. maclaganii* | PKLE-3 | MH397303.1 | Texas, USA | *Panicum virgatum* |
|  | 4 | *T. maclaganii* | KING-1 | MH397302.1 | Texas, USA | *Panicum virgatum* |
|  | 5 | *T. maclaganii* | KING-3 | MH397301.1 | Texas, USA | *Panicum virgatum* |
|  | 6 | *T. maclaganii* | TMPL-1 | MH397300.1 | Texas, USA | *Panicum virgatum* |
|  | 7 | *T. maclaganii* | TMPL-2 | MH397299.1 | Texas, USA | *Panicum virgatum* |
|  | 8 | *T. maclaganii* | Tm001NY09 | JF745116.1 | New York, USA | *Panicum virgatum* |
|  | 9 | *T. maclaganii* | 17-928 | MH256490.1 | Indiana, USA | *Panicum virgatum* |
|  | 10 | *T. maclaganii* | 17-994 | MH256491.1 | Indiana, USA | *Panicum virgatum* |
|  | 11 | *T. maclaganii* | 17-1055 | MH256492.1 | Indiana, USA | *Panicum virgatum* |
| *Tilletia nigrifaciens* Clade | 1 | *T. nigrifaciens* | Voucher BRIP 43865 | MH231796.1 | Queensland, Australia | *Phragmites australis* |
|  | 2 | *T. nigrifaciens* |  | AY309481.1 | Australia | *Phragmites australis* |
| *Tilletia ehrhartae* clade | 1 | *T. ehrhartae* | Voucher BRIP 28392 | MH231781.1 | Australia | *Ehrharta calycina* |
|  | 2 | *T. ehrhartae* | Voucher BRIP 28392 | AY770433.1 | Australia | *Ehrharta calycina* |
|  | 3 | *T. ehrhartae* | PREM 60989 | NR_132903.1 | South Africa | *Ehrharta calycina* Sm. |
| *Tilletia indica* clade | 1 | *T. indica* | Strain s001 | AF399890.1 | China | *Triticum aestivum* |
|  | 2 | *T. indica* | Strain s002 | AF399888.1 | China | *Triticum aestivum* |
|  | 3 | *T. indica* | Strain s003 | AF399889.1 | China | *Triticum aestivum* |
|  | 4 | *T. indica* | Strain s007 | AF398434.1 | China | *Triticum aestivum* |
|  | 5 | *T. indica* | DWR02 | KF945144.1 | Rajasthan, India | Wheat |
|  | 6 | *T. indica* | DWR03 | KF945145.1 | Haryana, India | Wheat |
|  | 7 | *T. indica* | DWR04 | KF945146.1 | Haryana, India | Wheat |
|  | 8 | *T. indica* | DWR08 | KF945150.1 | Haryana, India | Wheat |
|  | 9 | *T. indica* | DWR10 | KF945152.1 | UK, India | Wheat |
|  | 10 | *T. indica* | DWR11 | KF945153.1 | UP, India | Wheat |
|  | 11 | *T. indica* | KTi 19-2 | MT497986.1 | India | Wheat |
|  | 12 | *T. indica* | KTi 19-3 | MT497987.1 | India | Wheat |
|  | 13 | *T. indica* | KTi 19-4 | MT497988.1 | India | Wheat |
|  | 14 | *T. indica* | KTi 19-5 | MT497989.1 | India | Wheat |
|  | 15 | *T. indica* | KTi 19-7 | MT497991.1 | India | Wheat |
|  | 16 | *T. indica* | KTi 19-8 | MT497992.1 | India | Wheat |
|  | 17 | *T. indica* | KTi 19-9 | MT497993.1 | India | Wheat |
|  | 18 | *T. indica* | KTi 19-10 | MT497994.1 | India | Wheat |
|  | 19 | *T. indica* | KTi 19-12 | MT497996.1 | India | Wheat |
|  | 20 | *T. indica* | KTi 19-13 | MT497997.1 | India | Wheat |
|  | 21 | *T. indica* | KTi 19-14 | MT497998.1 | India | Wheat |
|  | 22 | *T. indica* | KTi 19-16 | MT498000.1 | India | wheat |
|  | 23 | *T. indica* | KTi 19-17 | MT498001.1 | India | Wheat |
|  | 24 | *T. indica* | KTi 19-19 | MT498003.1 | India | Wheat |
|  | 25 | *T. indica* | KTi 19-20 | MT498004.1 | India | Wheat |
|  | 26 | *T. indica* | KTi 19-21 | MT498005.1 | India | Wheat |
|  | 27 | *T. indica* | KTi 19-24 | MT498008.1 | India | Wheat |
|  | 28 | *T. indica* | KTi 19-25 | MT498009.1 | India | Wheat |
|  | 29 | *T. indica* | KTi 19-28 | MT498012.1 | India | Wheat |
|  | 30 | *T. indica* | KTi 19-29 | MT498013.1 | India | Wheat |
|  | 31 | *T. indica* | KTi 19-30 | MT498014.1 | India | Wheat |
|  | 32 | *T. indica* | KTi 19-31 | MT498015.1 | India | Wheat |
|  | 33 | *T. indica* | KTi 19-32 | MT498016.1 | India | Wheat |
|  | 34 | *T. indica* | Kti 19-33 | MT498017.1 | India | Wheat |
|  | 35 | *T. indica* | KTi 19-34 | MT498018.1 | India | Wheat |
|  | 36 | *T. indica* | KTi 19-36 | MT498020.1 | India | Wheat |
|  | 37 | *T. indica* | KTi 19-37 | MT498021.1 | India | Wheat |
|  | 38 | *T. indica* | KTi 19-38 | MT498022.1 | India | Wheat |
|  | 39 | *T. indica* | KTi 19-39 | MT498023.1 | India | Wheat |
|  | 40 | *T. indica* | KTi 19-40 | MT498024.1 | India | Wheat |
|  | 41 | *T. indica* | KTi 19-41 | MT498025.1 | India | Wheat |
|  | 42 | *T. indica* | KTi 19-44 | MT498029.1 | India | Wheat |
|  | 43 | *T. indica* | KTi 19-45 | MT498028.1 | India | Wheat |
|  | 44 | *T. indica* | KTi 19-46 | MT498030.1 | India | Wheat |
|  | 45 | *T. indica* | Strain DAOM 238027 | HQ317519.1 | Mexico | *Triticum* sp. |
|  | 46 | *T. indica* | Strain DAOM 238047 | HQ317581.1 | USA | *Triticum* sp. |
|  | 47 | *T. indica* | Strain DAOM 236409 | HQ317520.1 | India | *Triticum* sp. |
|  | 48 | *T. indica* | Strain BC 386 | AF310174.1 | USA | *Triticum aestivum* |
|  | 49 | *T. indica* | Strain BC 398 | AF310175.1 | USA | *Triticum aestivum* |
|  | 50 | *T. indica* | Strain BC 405 | AF310176.1 | USA | *Triticum aestivum* |
|  | 51 | *T. indica* | Strain LC 1012 | AF310178.1 | USA | *Triticum aestivum* |
|  | 52 | *T. indica* | PSWKBGH-1 | KX343896.1 | India | Wheat |
|  | 53 | *T. indica* | PSWKBGH-1-3 | KX343898.1 | India | Wheat |
|  | 54 | *T. indica* | RA-1 | KX369242.1 | UP, India | Wheat |
|  | 55 | *T. indica* | Karnal | AY560652.2 | India | Wheat |
|  | 56 | *T. indica* | Strain 2 | AF135434.1 | India | Wheat |
|  | 57 | *T. indica* | - | AY390350.2 | India | Wheat |
|  | 58 | *T. walkeri* | Strain BC447 | AF310185.1 | USA | *Lolium multiflorum* |
|  | 59 | *T. walkeri* | Strain 984 | JX126880.1 | China | Wheat |
|  | 60 | *T. walkeri* | Strain 160 | AF399887.1 | China | Wheat |
|  | 61 | *T. walkeri* | Strain LC_1123 | AF310180.1 | USA | *Lolium multiflorum* |
|  | 62 | *T. walkeri* | - | AF135435.1 | Oregon, USA | *Lolium multiflorum* |
| Out of group | 1 | *T. fusca* | Voucher WSP 71275 | EU257567.1 | Washington, USA | *Vulpia microstachys* |
|  | 2 | *T. fusca* | Strain 141 | AF310189.1 | USA | *Vulpia microstachys* |
|  | 1 | *T. brachypodii-mexicani* | Voucher HUV 16007 | MH231776.1 | Venezuela | *Brachypodium mexicanum* |
|  | 2 | *T. barclayana* | Strain 104 | AF399894.1 | China | *Pennisetum orientale* |
|  | 3 | *T. bromi* | Strain s023 | AF398461.1 | China | *Nardurus subulatus* |
|  | 4 | *T. challinorae* | Voucher BRIP 52502 | NR_119757.1 | USA | *Panicum trachyrhachis* |
|  | 5 | *T. lolioli* | Voucher WSP 71305 | EU257576.1 | USA | *Loliolum subulatum* |
|  | 6 | *T. narayanaraoana* | BRIP 47957 | GQ497894.1 | Australia | *Panicum trachyrhachis* |
|  | 7 | *Tilletia sp.* | ML-2012 Voucher KRAM 48771 | JQ793950.1 | Cameroon | *Urochloa maxima* (Jacq.) R. D. webster |

**SUPPLEMENTARY TABLE S2.** Sequence variation statistics of individual genes of the kernel smut fungal populations used in this study.

| **Sequence set** | **Diversity parameters** | | | | | **Neutrality test** | | | |
| --- | --- | --- | --- | --- | --- | --- | --- | --- | --- |
|  | **n** | **S** | **ND** | **θw** | **NM** | **Tajima’s D** | **Fu and Li’s D*** | **Fu and Li’s F*** | **R** |
| Elongation factor | 64 | 84 | 0.03 | 17.765 | 85 | 1.75 (NS)^a^ | 2.13 (*P*<0.02) | 2.41 (*P*<0.02) | 17 |
| RNA polymerase II | 64 | 189 | 0.116 | 39.97 | 205 | 3.13 (*P*<0.01) | 2.35 (*P*<0.02) | 3.23 (*P*<0.02) | 9 |
| LSU | 64 | 29 | 0.017 | 6.133 | 31 | 1.33 (NS) | 2.04 (*P*<0.02) | 2.14 (*P*<0.02) | 5 |
| ITS | 64 | 138 | 0.058 | 29.18 | 158 | -0.967 (NS) | 0.41 (NS) | 0.034 (NS) | 15 |

^a^NS=Not significant at *P=0.05*
