# Supplementary figures and images for: Multi-Locus Sequence Analysis Reveals Diversity of the Rice Kernel Smut Populations in the United States
Source: Front Microbiol. 2022 May 4;13:874120. doi: 10.3389/fmicb.2022.874120 (PMC9116506; doi:10.3389/fmicb.2022.874120)

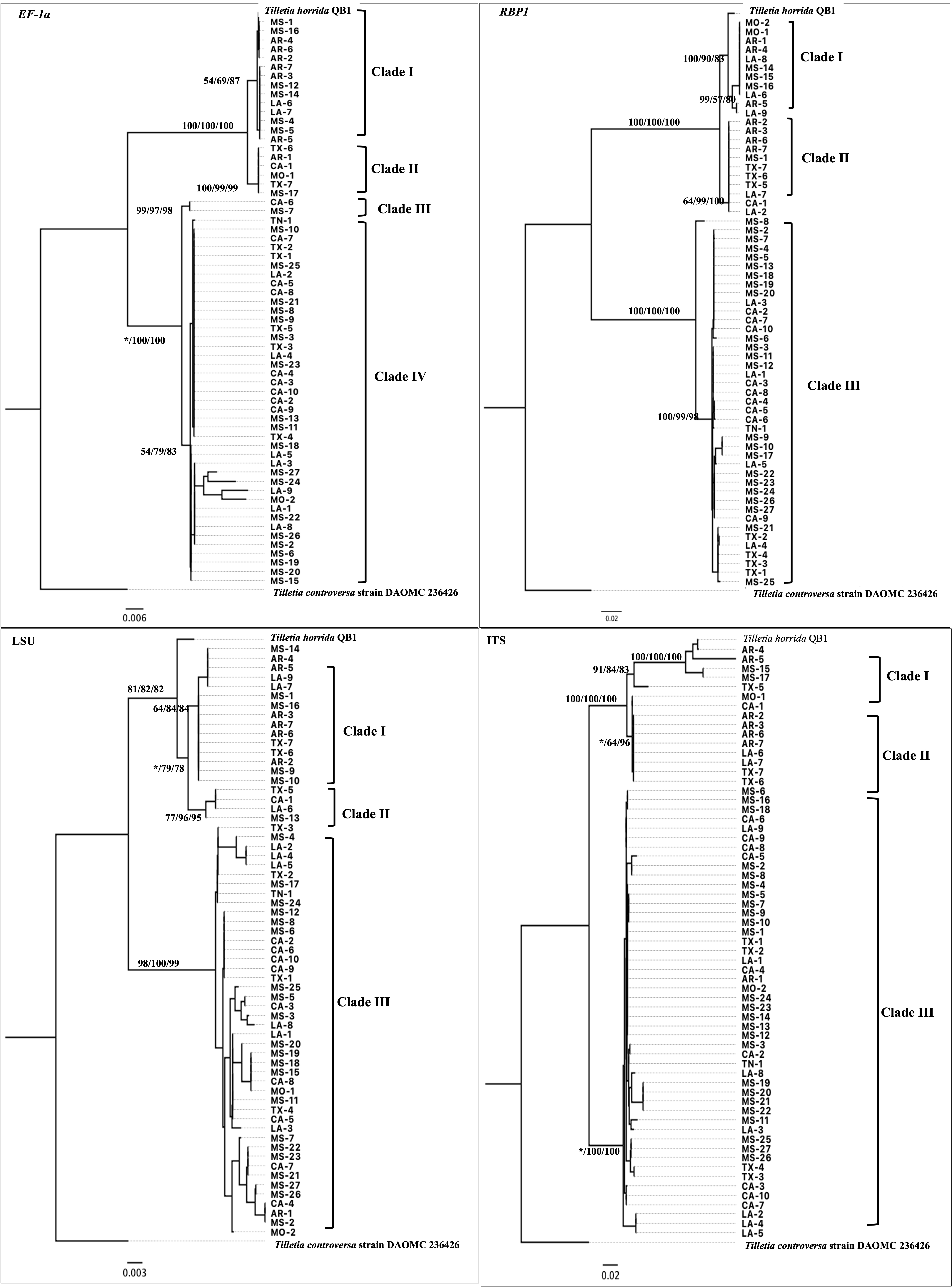

Supplement: Supplementary file 2 [file Image_1.JPEG]
